# A Splice Variant of ASC Regulates IL-1*β* Release and Aggregates Differently from Intact ASC

**DOI:** 10.1155/2009/287387

**Published:** 2009-09-15

**Authors:** Kazuhiko Matsushita, Michiko Takeoka, Junji Sagara, Naoki Itano, Yuka Kurose, Akihiro Nakamura, Shun'ichiro Taniguchi

**Affiliations:** ^1^Department of Molecular Oncology, Institute on Aging and Adaptation, Graduate School of Medicine, Shinshu University, Negano 390-8621, Japan; ^2^Department of Biomedical Laboratory Sciences, Shinshu University, Matsumoto 390-8621, Japan; ^3^Institute of Advanced Biosciences, Keio University, Tsuruoka 997-0017, Japan

## Abstract

The apoptosis-associated speck-like protein containing a caspase recruit domain (ASC) is involved in apoptosis and innate immunity and is a major adaptor molecule responsible for procaspase-1 activation. ASC mRNA is encoded by three exons: exons 1 and 3 encode a pyrin domain (PYD) and caspase recruit domain (CARD), respectively, and exon 2 encodes a proline and glycine-rich (PGR) domain. Here, we identified a variant ASC protein (vASC) lacking the PGR domain that was smaller than full length ASC (fASC) derived from fully transcribed mRNA and searched for differences in biochemical and biological nature. Both fASC and vASC were found to activate procaspase-1 to a similar degree, but the efficiency of IL-1*β* excretion was significantly higher for vASC. There was also a marked structural difference observed in the fibrous aggregates formed by fASC and vASC. These results suggest that although the PGR domain is dispensable for procaspase-1 activation, it plays an important role in the regulation of the molecular structure and activity of ASC.

## 1. Introduction

Cytosolic pathogen receptors and their downstream molecules have recently garnered attention in the field of inflammation and innate immunity. One such molecule, the apoptosis-associated speck-like protein containing a caspase recruit domain (ASC), is an adapter molecule consisting of a caspase recruit domain (CARD) and pyrin domain (PYD) that was first identified in our laboratory as a proapoptotic protein [1, 2]. The entire cDNA of ASC cloned by immunoscreening and RACE (rapid amplification of cDNA ends) has revealed that the open reading frame of 585 bp contains a PYD (1–90 amino acids) and a CARD (107–195 amino acids) [1]. According to GenBank accession number NP 037390, the cDNA of *ASC* consists of exon 1: 1–90 amino acids, exon 2: 91–110 amino acids, and exon 3: 111–195 amino acids. As for isoforms, an alternatively-spliced form of ASC lacking the PGR domain encoded by exon 2 of ASC was detected in IMR90 human diploid fibroblasts and 90SV fibroblasts by Conway et al. [3], and a short form of ASC (192 kDa) was found in THP-1 cells by Fernandes-Alnemri [4]. An ASC protein lacking 19 amino acids, which are encoded by exon 2 of ASC, is also reported in GenBank as isoform b (NP 660183). 

 ASC functions as a mediator in the cytosol-type inflammatory signaling pathway as well as a downstream molecule in Toll-like receptor signaling. ASC has been established to activate procaspase-1 [5] through its oligomerization in inflammasomes and process proIL-1*β* and proIL-18 into IL-1*β* and IL-18, respectively [6–8], leading to the establishment of innate immunity. Inflammasomes are formed by nucleoside oligomerization domain (NOD) like-receptor (NLR) family members, the adapter protein ASC, procaspase-1, and caspase-5 in some form of inflammasome [9]. Mutations in the basic components of inflammasomes appear to be responsible for several autoinflammatory diseases; mutations in the CIAS1 (cold induced autoinflammatory syndrome 1) gene encoding cryopyrin or in the MEFV (Mediterranean fever) gene encoding pyrin perturb the function of ASC and the consequent activation of procaspase-1 and IL-1*β* processing [10].

 ASC also plays an important role in an inflammatory form of cell death called pyroptosis [4, 11]. During pyroptosis, some ligands activate pyrin by unmasking its PYD in the pyroptosome, thereby allowing it to interact with ASC and facilitating ASC dimerization into an active ASC pyroptosome that activates caspase-1 [12]. However, the PYD of pyrin is reported to interfere with the ability of cryopyrin to associate with ASC, indicating negative regulation of ASC proximity in inflammasomes [13]. Since the oligomerization of ASC clearly plays a pivotal role in innate immunity and pyroptosis to activate caspase-1 but the function of the PGR domain remains to be clarified, we investigated the regulation of molecular structure and activity of a variant form of ASC (vASC) with a missing PGR domain in comparison with that of full length ASC (fASC).

## 2. Materials and Methods

### 2.1. Detection of an ASC Variant (vASC) In HL-60 Cells by Mass Spectrometry

HL-60 cells were cultured in RPMI 1640 supplemented with 10% fetal bovine serum. ASC and an unidentified Protein X separated by SDS–PAGE using antihuman ASC monoclonal antibody (MBL, Nagoya, Japan) were excised from a gel after staining with Coomassie brilliant blue R-250 and digested using a Montage In-Gel Digest_96_ Kit (Millipore.com, Massachusetts, USA) according to the manufacturer's protocol. The purified peptides were then analyzed with *α*-CHCA (cyano-4-hydroxycinnamic acid) by MALDI-TOF MS. A Voyager Elite XL Biospectrometry workstation (PerSeptive Biosystem, Framingham, MA) equipped with a delayed extraction ion source was used for MALDI-TOF MS analysis in the reflector mode with the following parameters: accelerating voltage, 22 kV; grid voltage, 80% of accelerating voltage; guide wire voltage, 0.05% of accelerating voltage; and delay time, 200 nanoseconds. A nitrogen laser (337 nm) was used for ionization. Calibration was performed using the monoisotopic peaks of the *α*-CHCA dimer (379.0930 m/z), angiotensin I (1296.6853 m/z), and ACTH (7–38 clip) (3657.9294 m/z).

### 2.2. Preparation of Expression Vectors

The *cDNAs* of human fASC, vASC*, procaspase-1,* and *pro1L-1β* were prepared from HL-60 mRNA by RT-PCR (RNA LA PCR Kit, Takara Bio, Otsu, Japan) and cloned into pcDNA3 vectors (Invitrogen, Carlsbad, CA, USA).

### 2.3. Western Blotting and Immunostaining

The prepared *procaspase-1* and *proIL-1β* and even* fASC* or *vASC* expression vectors were cotransfected into Cos7 cells using FuGENE6 Transfection Reagent (Roche, Mannheim, Germany) for transient gene-expression. The cells were dissolved with 1% SDS 24 hours after transfection, and then samples were loaded onto an SDS-PAGE 6–15% polyacrylamide gel, transferred to PVDF membranes, and developed by ECL (GE Healthcare, Buckingham, UK). Antibodies of Caspase-1 and IL-1*β* were obtained from Santa Cruz (California, USA). For cellular immunostaining, cells were fixed in a 37% formaldehyde solution, stained with Cy3-ASC antibody and Hoechst 3342, and observed with an immunofluorescense microscope (Axiovert S100, Carl Zeiss, Tokyo, Japan).

### 2.4. Measurement of Excreted IL-1*β*

IL-1*β* excreted by Cos7 cells cotransfected with expression—vectors (30 ng *procaspase-1* and 100 ng *proIL-1β* with 50 ng *fASC or vASC*) was measured 24 hours after plating using a Human IL-1*β* immunoassay kit (R and D systems, Minneapolis, USA) according to manufacturer's instructions. 

### 2.5. Statistical Analysis

Analysis of variance was performed and the differences between groups in each set were evaluated by Fisher's protected least significant difference test using Stat View 5.0 software (SUS Institute, Inc., Berkley, CA, USA). All values were expressed as mean ± standard error (SE) of the mean. A *P* value of less than .05 was considered significant.

## 3. Results

### 3.1. Analysis of Protein X by Mass Spectrometry

**Figure 1:**
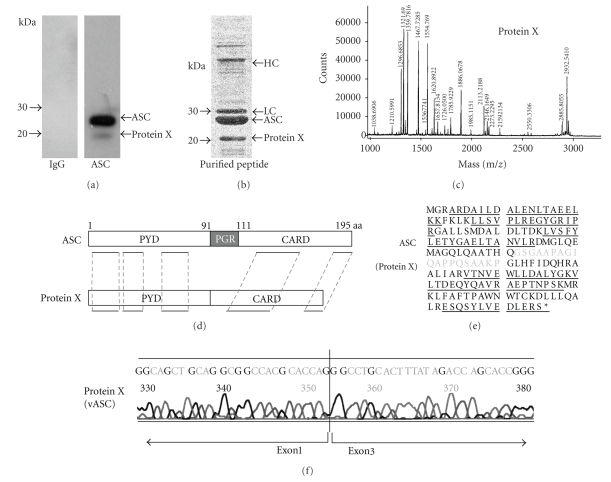
Analysis of protein X by mass spectrometry*.* (a) HL-60 cells were subjected to SDS-PAGE and western blot analysis using antihuman ASC antibody. In addition to full length ASC (fASC), an additional polypeptide of 20 kDa (Protein X) was detected. (b) Proteins that were affinity-purified using anti-ASC antibody were stained with Coomassie Blue. HC: Ig heavy chain, LC: Ig light chain. (c) Mass spectrum of digested protein X is shown. (d) Structures of ASC and Protein X. Homologous regions are underlined. aa: amino acid. (e) Amino acid sequence of fASC from a database (GenBank Accession no. NP 037390) and Protein X is displayed. Homologous sequences are underlined. The amino acid sequence written in light characters was not detected in Protein X. (f) Sequence of ASC cDNA extracted from HL-60 cells as Protein X is shown.

When HL-60 cells were subjected to SDS-PAGE, anti-ASC antibody detected one band of 23 kDa, which corresponds to fASC, and another of 20 kDa, called Protein X (Figure 1(a), right lane). A mouse IgG control is shown in Figure 1(a), left lane. Affinity-purified polypeptides from HL-60 cell lysates incubated with anti-ASC antibody are displayed in Figure 1(b). The unknown protein X was then treated by trypsin ingel digestion and analyzed with MALDI-TOF MS (Figure 1(c)), and peptide mass data were subjected to a database search using MS-Fit, a peptide mass fingerprinting program from the University of California, San Francisco. Since the N and C terminal sequences of protein X were confirmed to be the same as fASC but lacked the amino acid sequence corresponding to the PGR domain (Figures 1(d) and 1(e)), we were able to identify protein X as vASC lacking a PGR domain. The sequence of *vASC* cDNA from mRNA extracted from HL-60 cells is displayed in Figure 1(f) and confirms deletion of exon 2 from fASC. This vASC corresponds to a protein reported in the GeneBank as association number NP 660183. Thus, protein X was not a degraded ASC but rather a variant form of ASC derived from alternatively spliced mRNA missing exon 2 of the *ASC* gene.

**Figure 2:**
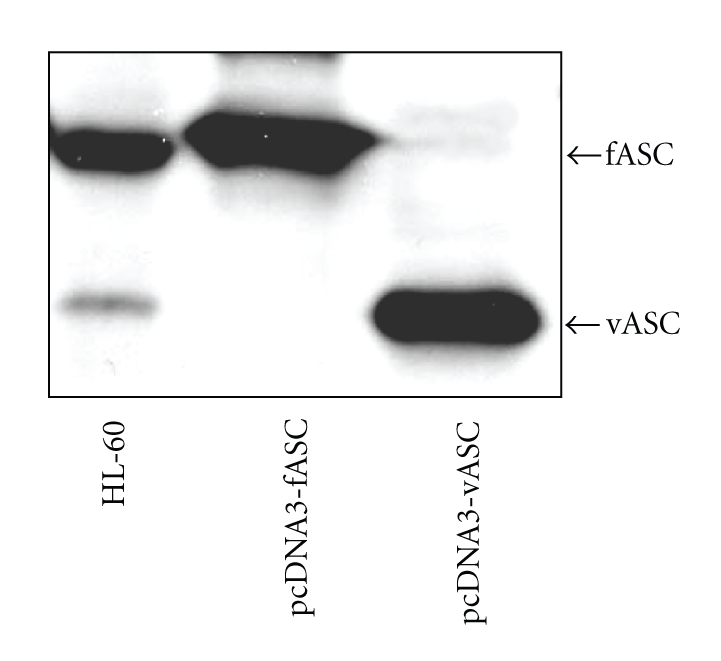
Molecular size comparison of endogenous (HL-60) and exogenous (Cos7) fASC and vASC proteins. Western blotting of endogenous fASC and vASC in HL-60 cells and exogenously expressed products of pcDNA3 fASC and pcDNA3 vASC in Cos7 cells. The band of fASC is 23 kDa and that of vASC is 20 kDa.

 Next, *fASC* or *vASC* cDNA expression vectors were transfected into Cos7 cells and, 23 kDa and 20 kDa proteins were induced, respectively (Figure 2). Both of these exogenously expressed proteins were the same size as the endogenous ASC-related products found in HL-60 cells seen in PAGE (Figure 1(a)). Since the Cos7 cells used in transfection experiments were derived from African Green Monkey kidney fibroblasts, endogenous expression of fASC or vASC was not detected in Cos7 cells by antihuman ASC antibody.

### 3.2. ASC-Dependent Activation of Procaspase-1 and IL-1*β*

**Figure 3:**
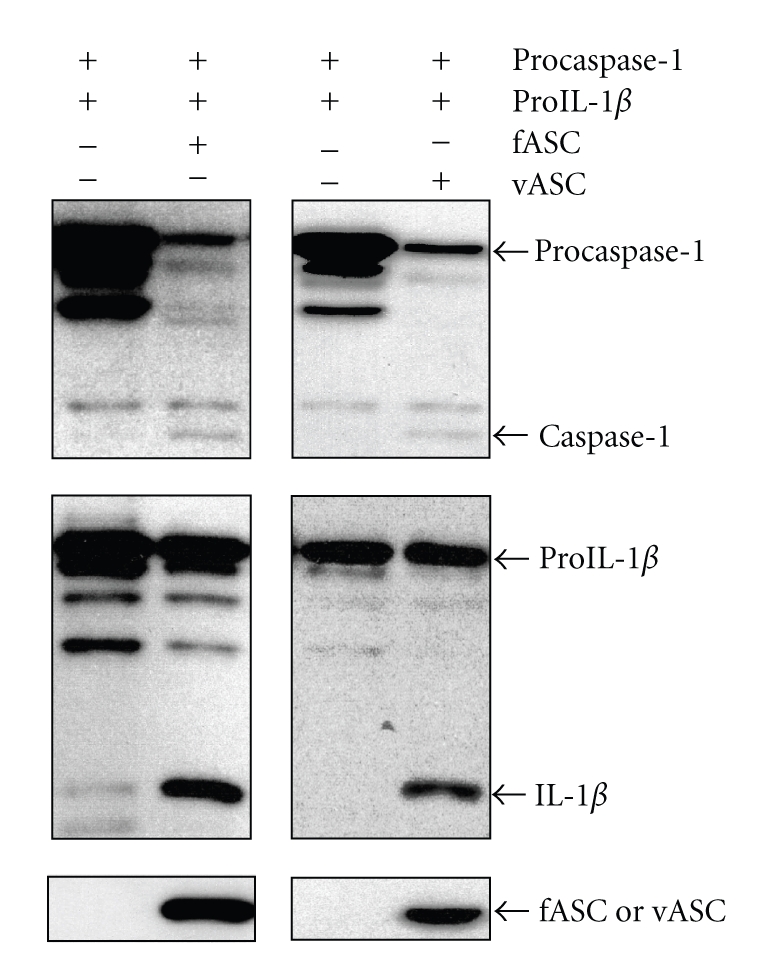
Comparison of caspase-1 and IL-*β* activation between fASC and vASC. Western blotting of coexpressed procaspase-1 and proIL-1*β* with fASC or vASC in Cos7 cells. Procaspase-1: 45 kDa, caspase-1 (active form): 20 kDa, proIL-1*β*: 31 kDa, IL-1*β*(active form): 17 kDa.

**Figure 4:**
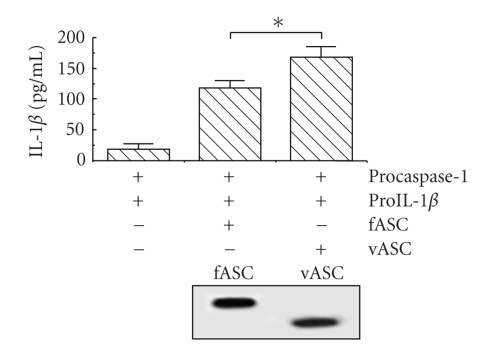
Comparison of excreted IL-1*β* levels. IL-1*β* in the culture medium of Cos7 cells cotransfected with expression vectors (*procaspase-1* and *proIL-1β* with fASC or vASC) was measured by ELISA. Results are shown as mean ± SE.*n* = 3, *P < .05. Expression levels of fASC and vASC are shown under the graph.

When the expression vectors of *procaspase-1* and, *proIL-1β* with *fASC* or *procaspase-1* and *proIL-1β* with *vASC* were expressed in Cos7 cells, the 45 kDa band of procaspase-1 decreased in both cases while the activated 20 kDa form appeared (Figure 3). The expression level of caspase-1 versus fASC in fASC-transfected cells measured using NIH imaging from triplicate experiments was not significantly different from that of vASC, indicating that caspase-1 was activated by both fASC and vASC at comparable levels (Figure 3). Similar findings were observed between the two proteins for the 31 kDa band of pro IL-1*β* and the 17 kDa-activated form of IL-1*β*. Additionally, the expression level of IL-1*β* versus fASC in fASC-transfected cells measured using NIH imaging from triplicate experiments was not significantly different from that of vASC (Figure 3). However, since the level of active IL-1*β* as detected by western blotting was relatively low, we suspected that a large amount of active IL-1*β* could have been excreted from the cells. Surprisingly, when we examined IL-1*β* levels in the medium, the amount of IL-1*β* was significantly higher in the vASC transfectants than in the fASC transfectants (*n* = 3, *P* < .05, Figure 4). Representative western blotting data of fASC and vASC from three experiments are displayed in Figure 4, under the graph. Although protein levels of vASC were smaller than those of fASC, the amount of released IL-1*β* was higher.

### 3.3. Intracellular Distribution of fASC and vASC

**Figure 5:**
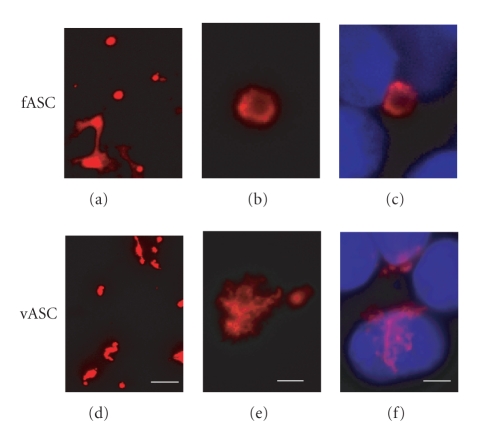
Intracellular distribution of fASC and vASC in Cos7 cells. Expression vectors of fASC or vASC were transfected into Cos7 cells. (a), (d): aggregation of fASC and vASC, scale bar: 30 *μ*M. (b), (e): magnified pictures of (a) and (d), scale bar: 5 *μ*M. (c), (f): aggregations with nuclei, scale bar: 5 *μ*M.

The distribution of exogenously transfected gene products was examined in Cos7 cells. Both products were seen to form fibrous aggregates (Figure 5), but fASC formed typical circular fibrous aggregates whereas those of vASC were branched; almost 40% of fASC-expressing cells exhibited a diffused fASC pattern and 60% of cells formed circular fibrous aggregates, but vASC formed branched fibrous aggregates in all cells expressing it.

## 4. Discussion

The homophilic interactions of the *N*-terminal PYD and *C*-terminal CARD domains have been revealed by immunoprecipitation to be involved in the self-association and filament-like aggregation of ASC [14]. PYD has been shown to mediate the self-association of ASC [15]. ASC and PYD-family proteins seem to interact via their PYD domain [16], while the CARD domain of ASC has been shown to bind to the CARD domain of caspase-1 [1, 17, 18]. Ectopic expression of full-length ASC, but not its isolated CARD or PYD domains, with procaspase-1 previously induced activation of procaspase-1 and processing of proIL-1*β* in transfected cells. Thus, PYD functions as the oligomerization domain, and CARD functions as the effector domain of ASC in the caspase-1 activation pathway [18]. In spite of these discoveries, the role of the PGR domain, which is located between PYD and CARD, remains unclear.

 Our results showed no discernable differences in caspase-1 activation between fASC and vASC. However, the levels of excreted IL-1*β* in the medium was significantly higher in vASC transfectants than in fASC transfectants, and immunostaining revealed remarkable differences in aggregate formation and ASC distribution.

**Figure 6:**
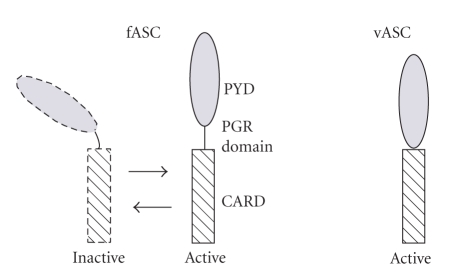
Hypothesized structure of fASC and vASC showing the PGR domain hinge.

 The PGR domain contains three glycine and four proline residues. Glycine is known to confer flexibility to protein structures [19], making them candidates to act as movable hinges. Proline motifs are also found in molecular hinges, and *α*-helice conformational changes can be generated by a proline-hinge in transmembrane proteins [20, 21]. Thus, the PGR domain may impart a bending property to ASC and can be considered to be a hinge between the PYD and CARD domains that is folded in the inactive form of ASC (Figure 6). Our results with the “hingeless” vASC support this notion in that unfoldable ASC may have been constitutively activated and formed branched fibrous aggregates in cells expressing the product. 

 In terms of IL-1*β* released, the preventative effects of glycine on proIL-1*β,* but not IL-1*β*, released from macrophages [22] and regulation by Ca^2+^ on IL-1*β* release from lysosomes via exocytotic fusion [23] have been reported. The involvement of fASC or vASC in the release of IL-1*β* is unclear, however. Together with a strong electric dipole moment of the PYD domain of ASC [24], our present results suggest that the PGR domain may affect the 3-dimensional structure of ASC and thus play an important role in the activation of ASC.

## 5. Conclusion

vASC lacking the PGR domain excreted higher levels of IL-1*β* and formed a more fibrous aggregation than fASC. We hypothesize that this domain plays the structural role of a hinge between PYD and CARD domains. fASC may be in an inactive folded state in the absence of ligands, whereas vASC may be in a permanently active state.
